# Basilar Invagination With Chiari Type I Malformation and Atlanto-Axial Instability: A Rare Case Report

**DOI:** 10.7759/cureus.44141

**Published:** 2023-08-26

**Authors:** Nandini Sanjay, Chandan Y.S, Krishan Yadav, Anees Dudekula

**Affiliations:** 1 Orthopaedics, Sri Devaraj Urs Academy of Higher Education and Research, Kolar, IND; 2 Neurosurgery, Sri Devaraj Urs Academy of Higher Education and Research, Kolar, IND; 3 Radiodiagnosis, Sri Devaraj Urs Academy of Higher Education and Research, Kolar, IND

**Keywords:** cranio-vertebral junction, atlantoaxial instability, atlanto-axial fusion, chiari malformation, basilar invagination

## Abstract

Basilar invagination (BI) and Chiari malformation type I (CM-I) are important anomalies involving the craniovertebral junction (CVJ) involving the skull base and occipitocervical region. The incidence of BI is rare involving < 1% of the general population worldwide. They present with varied and complex clinical-radiological features. We present a 36-year-old male who displayed complaints of persistent reeling sensation at our center. Clinical examination revealed bilateral cerebellar signs along with nystagmus and restricted neck movements. Imaging revealed evidence of BI with cerebellar tonsil herniation of _~_14.7 mm. Atlantodens interval of 6 mm was noted. The unexpected findings of C1-C2 fusion and instability were also noted. We describe a rare case of BI with C1 prolapse into the foramen magnum along with CM-1 malformation and congenital fusion of C1-C2. We conclude that the treatment algorithm for these rare cases is not very well established and is individually dependent.

## Introduction

Chiari malformations (CMs) - a part of hindbrain herniation syndromes - are a group of rare congenital disorders that occur when part of the brain and its membranes herniate via foramen magnum. “Professor Hans Chiari (1851-1916),” a pathologist in Prague, Czech Republic, described four different types of CMs in the late eighteenth century. Chiari type I (CM-I) and II (CM-II) are the most commonly found.

Type I lesion is caused by cerebellar tonsils' caudal descent. An elongated fourth ventricle may sometimes be noted [1]. A herniation of more than 3 mm on sagittal MR imaging is considered abnormal. Type II lesions consist of caudal descent of cerebellar vermis, lower brain stem, and fourth ventricle, which are usually accompanied by myelomeningocele [1].

Basilar invagination (BI) is a condition, mostly degenerative or congenital, where the odontoid process of axis bone protrudes into the foramen magnum with posterior displacement of the bulb leading to small posterior fossa [2].

The association between CM-I and BI is thought to be associated with abnormalities in the development of the upper cervical spine and skull. While these conditions can occur independently, their coexistence can lead to more severe symptoms and complications. Cerebellar BI is usually considered a congenital condition but may be developed in rare instances as a result of rheumatoid arthritis (RA), Paget’s disease, tumors of cervical spine, trauma, etc. [3].

In this report, we show a rare case of congenital BI having cerebellar tonsillar herniation who also had associated atlantoaxial joint instability.

## Case presentation

History

A 32-year-old male presented to the emergency department having problems with reeling sensation and imbalance while walking for three months. There was no history of headache, vomiting, or seizures.

Recently, the patient has been diagnosed with diabetes mellitus type II along with hypertension, which was well controlled with regular medication. There was no significant past or family history.

Examination

Skull and spine examination revealed a short neck with global restriction of neck movements. Neurological evaluation revealed a conscious and well-oriented patient with normal speech and no cranial nerve deficit. There were mild bilateral cerebellar signs including nystagmus along with a positive Romberg’s sign. There were no focal limb neurological deficits.

Investigations

**Figure 1:**
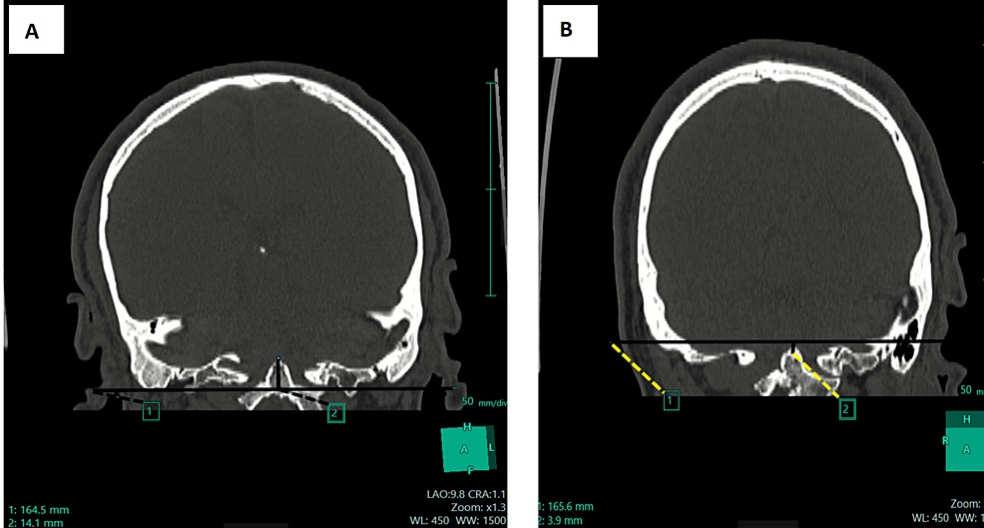
Coronal CT images show that the tip of the odontoid process is 14.1 mm above the bimastoid line (A). The tip of the odontoid process is at a distance of 3.9 mm from the digastric line (B).

**Figure 2:**
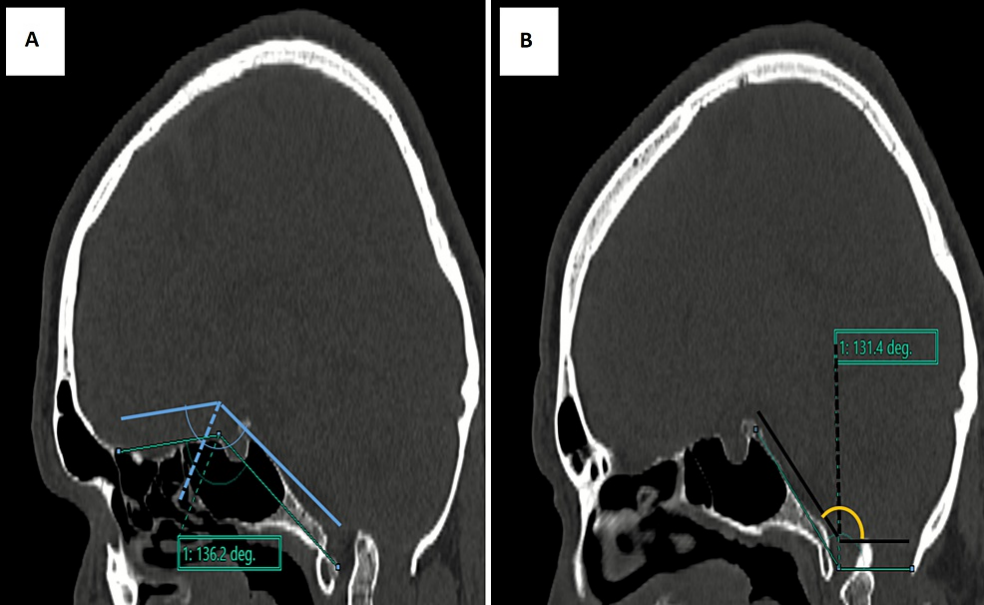
Sagittal CT images show Welcher's basal angle measuring 136.2 degrees (within normal limits) (A) and Boogard’s angle measuring 131.4 degrees (B)

**Figure 3:**
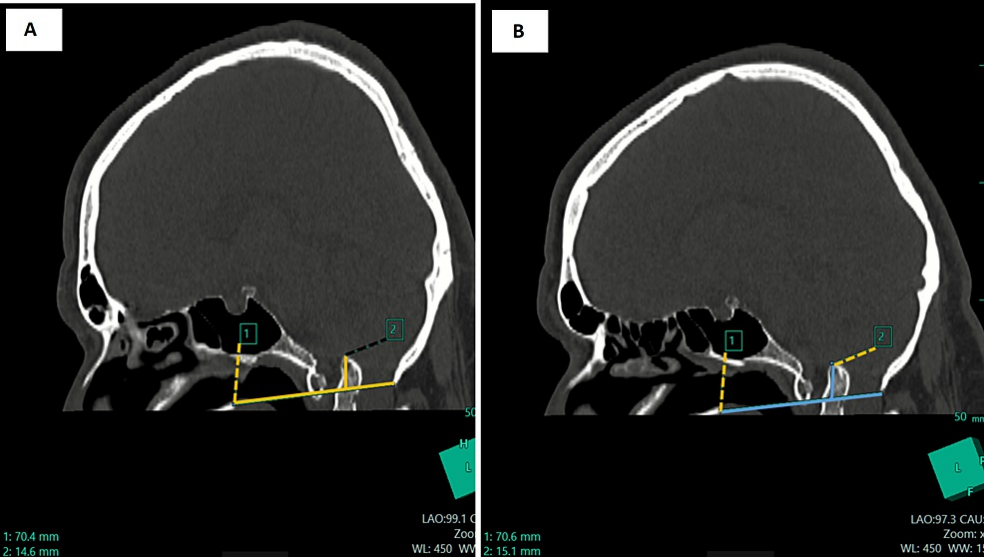
Sagittal CT images show that the Chamberlain line, tip of the odontoid process, is ~14.6 mm above this line (A) and the McGregor’s line, tip of the odontoid process is ~14.8 mm above this line (B)

Digital radiographs of the neck and CV junction revealed a fusion of the atlas (C1) and axis (C2). Craniometry revealed the tip of the odontoid process above McRae’s (7.5 mm) and Wackenheim’s line. The height index of Klaus was decreased (27.1 mm). Coronal CT images showed the odontoid process tip located above the bimastoid line and 3.9 mm from the digastric line (Figure 1, Panels A and B). Skull base angles were within normal measurements (Figure 2, Panels A and B). Sagittal CT images showed abnormal Chamberlain’s and McGregor’s lines (Figure 3, Panels A and B). An atlantodens interval of 6 mm was noted.

**Figure 4:**
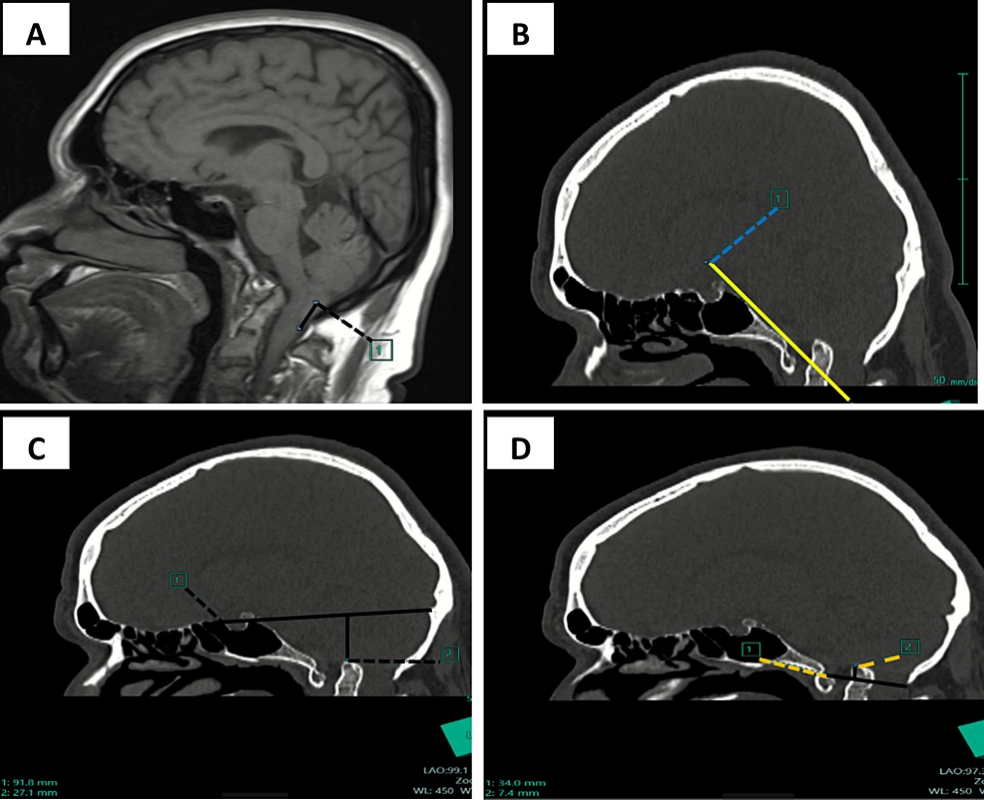
MRI showing caudal herniation of bilateral cerebellar tonsils noted for a distance of ~14.7 mm below the foramen magnum (A). Sagittal CT images show that Wackenheim’s line is intersecting the odontoid process (B). The height index of Klaus, which is the distance between the tip of the dens and the tuberculum torcula line, is 27.1 mm (C), McRae line, which is the tip of the odontoid process is ~7.5 mm above the line (D).

MRI brain and cervical spine revealed caudal herniation of bilateral cerebellar tonsils (a distance of 14.7 mm below the foramen magnum) (Figure 4, Panels A-D). The tested laboratory parameters comprising complete blood cell counts, comprehensive metabolic panel, and blood clotting function were all found to be normal.

Operative management

**Figure 5:**
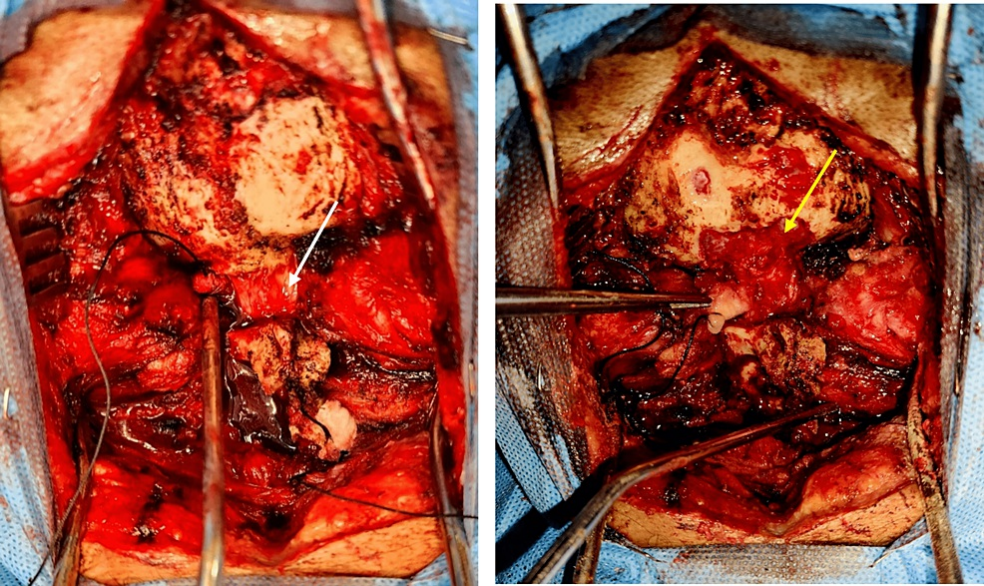
Cranial-caudal, intraoperative image: The left image shows the congenital band (white arrow), and the right image shows the foramen magnum decompression (yellow arrow)

**Figure 6:**
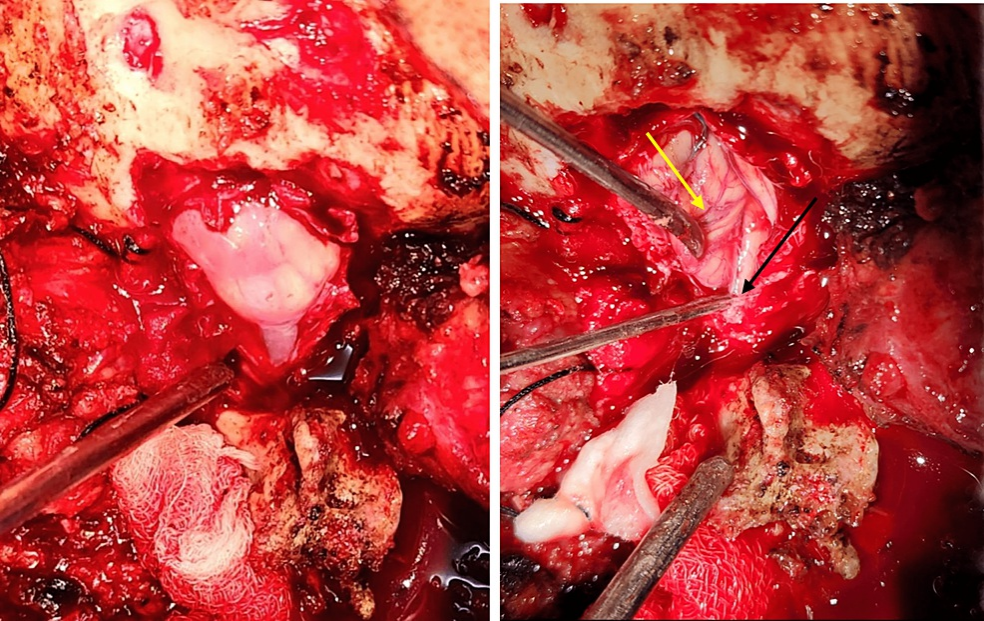
Cranial-caudal, intraoperative image: The left image shows the cisterna magna after opening the dura, and the right image shows the herniation of cerebellar tonsil (yellow arrow) compressing on the spinal cord (black arrow).

**Figure 7:**
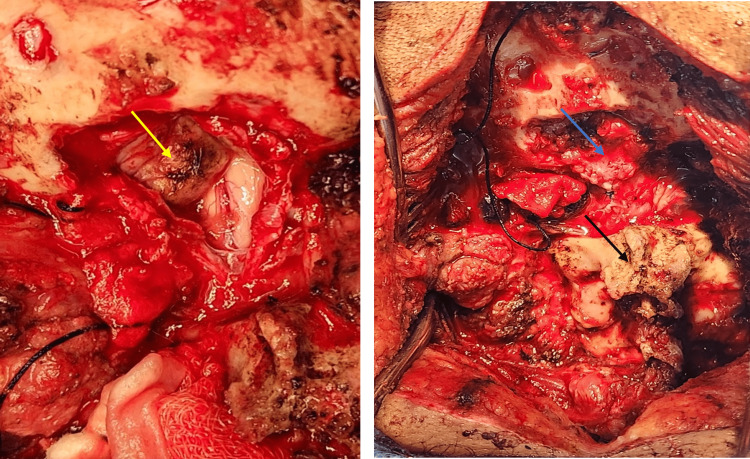
The left image shows the left herniated cerebellar tonsil after cauterization, and the right image depicts the following duroplasty (blue arrow) showing the fusion of C1-C2 spinous process (black arrow).

**Figure 8:**
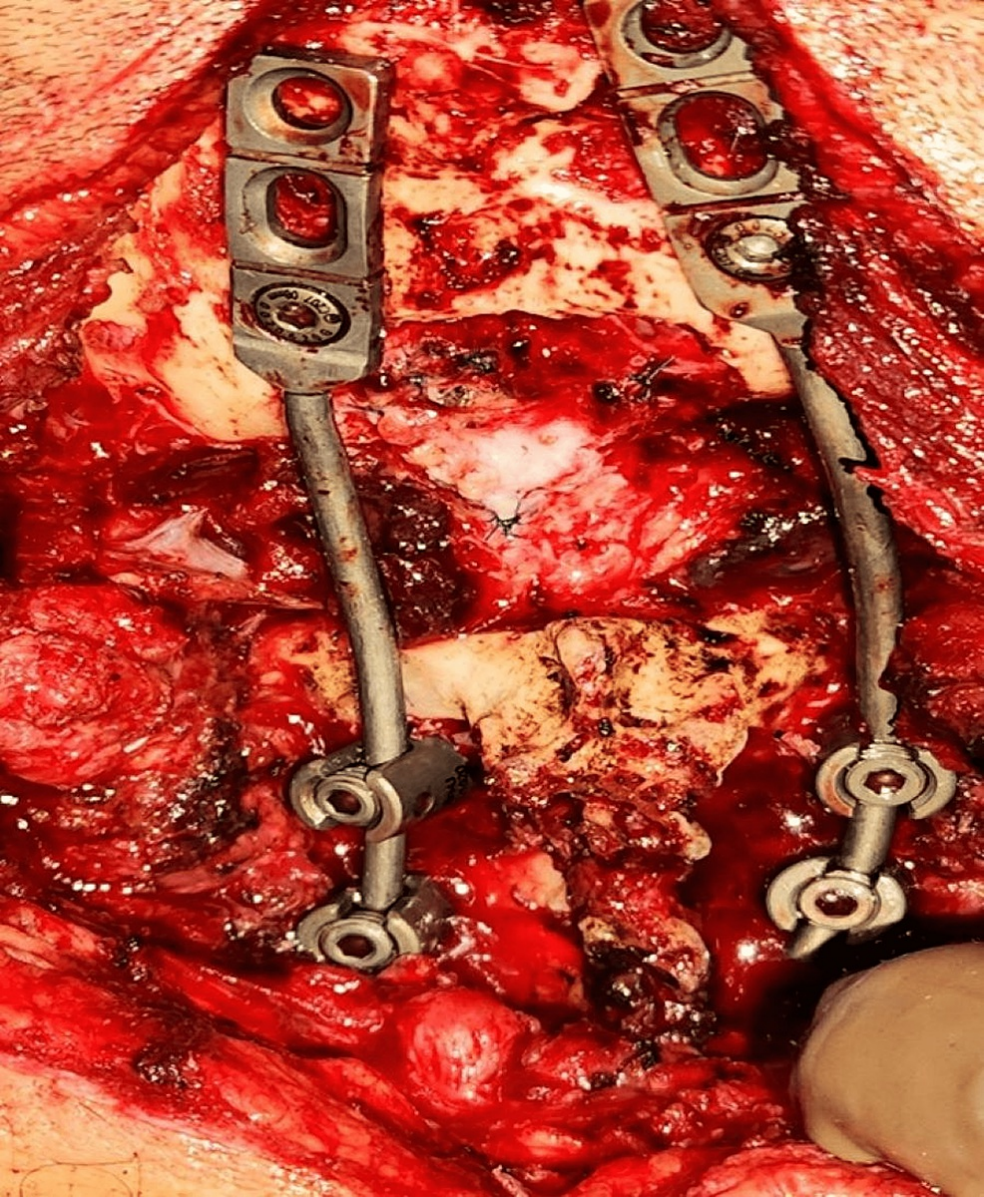
Intraoperative image showing the occipitocervical fusion with occipital screws, C2-C3 screws, and rods

Biaxial skull traction was tried intraoperatively, but reduction could not be obtained. The patient then underwent foramen magnum decompression, excision of a thick band, cauterization to shrink the herniated cerebellar tonsils, and lax duroplasty using fascia lata. Thereafter, “C2 pedicle screws & C3 lateral mass screws” have been positioned, and fixation along with occipital-cervical fusion was done (Figures 5-8).

The postoperative phase was uneventful, and the patient's symptoms improved. At discharge, the patient was instructed to continue the three-month usage of the Philadelphia collar.

## Discussion

BI is a developmental defect characterized by various symptoms such as headache, neck stiffness, neck pain, diplopia, rhinolalia, dysphagia, paresthesia of limbs and face, vertigo, and progressive cerebellar ataxia.

Although the exact etiology of BI is not known, it has been mostly a result of a few factors together. There have been studies and debates about whether BI must be considered a radiographic finding only, so various radiological parameters have been made to help in its diagnosis. Most of the BI patients also have platybasia, atlantooccipital assimilation, or atlas hypoplasia [4]. Our patient did not have platybasia as per the craniometry and measurement of skull base angles.

Chiari identified variations in the inferior position of cerebellar tonsils and stated that a cerebellar herniation of 3-5 mm is abnormal; this condition is called CM-I. In a few cases, relationships with other disorders of the craniocervical junction, most notably BI, complicate CM-I [5]. In our patient, the cerebellar tonsillar herniation was very significant (approximately 14.7 mm).

Goel discussed two classification systems in relation to BI. The first classification divides BI into two groups based on the absence/presence of CM, respectively. The presence or absence of demonstrable instability of the atlantoaxial area, which has been evidenced by the odontoid process distancing from the atlas anterior arch, was the basis of the second classification. The most common BI lesion was an odontoid prolapsing abnormally into FM with atlantoaxial dislocation (group A). The primary pathogenetic factor in group B patients seemed to be a decrease in posterior cranial fossa volume without herniation of the odontoid process [6]. Our patient can be classified under Group II A since he had atlantoaxial instability along with reduced posterior fossa volume due to BI and CM.

The measured caudal dislocation of cerebellar tonsils is essential for diagnostic purposes. Nevertheless, the tonsillar migration’s standard limit remains disputed, with the normal limit established as a distance between 3 and 5 mm, depending on age. In our study, the patient had a migration of 14.7 mm below the foramen magnum as seen in Figure 4. Chamberlain [7] proposed a method for preoperative diagnosis depending on the breach of the skull baseline as suggested by him. CT images in the studied patient revealed that the odontoid process’ tip was 14.6 mm above this line as shown in previous images.

The majority of group B disease patients had the odontoid process tip below Wackenheim's clivus line and McRae's foramen magnum line [8,9]. In the above case, however, the odontoid process was discovered to be 7.5 mm above both McRae's line and Wackenheim's clivus line, as shown earlier. This type of BI was uncommon, and treating it was challenging because of the complicated anatomical and kinematic interactions at the junction of the cranial vertebra.

Surgery for group A of BI is standardized; however, group B of BI therapy is still up for debate. The majority of researchers concur that FM decompression is necessary to expand the posterior cerebral fossa in group B BI, although some also think that craniovertebral stabilization in addition to FM decompression is a workable therapy option [10,11]. The above type of BI was uncommon, and treating it was challenging because of the complicated anatomical and kinematic interactions at the junction of the cranial vertebra.

According to a report by Phillips [12] in 1955, attempts at conservative treatment did not produce convincing results. Brito et al. reported in their research on atlantoaxial instability in rheumatoid inflammatory processes that decompression with posterior fixation is necessary; hence, they suggested this approach in BI for joint instability [13].

Priority is given to anterior decompressive surgery, which entails odontoid apophysis resection via a transoral route when the atlantoaxial joint with odontoid process protrusion and brainstem compression are deemed irreducible or fixed in a BI patient [11,14].

Menezes et al. offered recommendations for the surgical treatment of craniocervical anomalies after considering various kinds of compression and the possibility of reduction. The treatment protocol for irreducible or fixed defects depends on the position of compression in dorsal and ventral groups, and posterior fixation was advised for reducible craniovertebral abnormalities [15].

In 2005, Kassam et al. [16] carried out an endoscopic transnasal odontoidectomy and showed it was feasible. Since the advent of endoscopic technology, this strategy has been utilized to treat BI with cervicomedullary compression successfully [17].

In his recent studies, Goel has proposed newer treatment strategies involving craniovertebral realignment and arthrodesis [18]. As a result of these findings, he has reaffirmed the instability theory, thereby indicating that in all these cases, foramen magnum decompression is not required, and in fact, it would not have a long-term effect.

## Conclusions

BI with CM type 1 is an uncommon entity, and treating this condition can be challenging. According to the available literature, the surgical management of these cases is either by posterior or anterior approach. The treatment of this patient was challenging as this condition was also associated with C1-C2 fusion along with instability.

We have managed this case by posterior decompression and occipital-cervical fusion. Earlier, some authors have advocated a transoral approach for these conditions. We propose that the treatment of these rare presentations should be individualized based on the clinical condition of the patient, symptomology, craniometry, and intraoperative findings as well as the surgeon’s preference. Although the result of the above case was satisfactory, a better understanding and planning to treat this rare case, a longer follow-up period, and a larger number of patients as well as collaboration of multiple centers are required.
